# Personalized Course Recommendation System Fusing with Knowledge Graph and Collaborative Filtering

**DOI:** 10.1155/2021/9590502

**Published:** 2021-09-25

**Authors:** Gongwen Xu, Guangyu Jia, Lin Shi, Zhijun Zhang

**Affiliations:** ^1^School of Business, Shandong Jianzhu University, Jinan 250101, China; ^2^School of Computer Science and Technology, Shandong Jianzhu University, Jinan 250101, China

## Abstract

Personalized courses recommendation technology is one of the hotspots in online education field. A good recommendation algorithm can stimulate learners' enthusiasm and give full play to different learners' learning personality. At present, the popular collaborative filtering algorithm ignores the semantic relationship between recommendation items, resulting in unsatisfactory recommendation results. In this paper, an algorithm combining knowledge graph and collaborative filtering is proposed. Firstly, the knowledge graph representation learning method is used to embed the semantic information of the items into a low-dimensional semantic space; then, the semantic similarity between the recommended items is calculated, and then, this item semantic information is fused into the collaborative filtering recommendation algorithm. This algorithm increases the performance of recommendation at the semantic level. The results show that the proposed algorithm can effectively recommend courses for learners and has higher values on precision, recall, and F1 than the traditional recommendation algorithm.

## 1. Introduction

Recently, with the integration of Internet and education, various online education platforms have emerged and developed rapidly. These platforms have accumulated many users with their high-quality and massive resources. Especially affected by the COVID-19, the online education all over the world has developed rapidly. The daily active users of Coursera, edX, XuetangX, and other online education platforms have reached more than 10 million. The explosive growth of online education has changed the traditional teaching mode and made it a reality for people to learn anytime and anywhere. Online education has become an important educational model and technical way for learners to acquire knowledge and expand skills and academic education [1].

However, nowadays a wide range of learning directions and all kinds of courses have sprung up on the online education platform. The massive courses also bring confusion to learners and have some disadvantages for learners. Firstly, it is difficult for learners to find suitable courses in a short time; secondly, the learning route that learners are interested in is complex, while the courses in the school training program tend to be single and repetitive, so the online learners' satisfaction is low and cannot achieve the expected teaching effect; thirdly, most online education platforms do not provide the same effective learning guidance and curriculum planning for learners as the traditional education. Learners are usually short of an in-depth understanding of the overall knowledge structure, while the number of Internet learning resources is miscellaneous, so learners fall into a large number of course choices, resulting in information overload and even the low course passing rate [2, 3].

How to provide personalized content for learners in massive courses resources is a problem worthy of research. Mining the interests of learners on the online education platform would help to better understand the needs of learners and help the platform provide personalized teaching services for learners. As the solution to this problem, the traditional interest mining methods either take the courses that learners are interested in as the interest point, or take the topic of the discussion area as the interest point. So many online education teams at home and abroad are also discussing and studying this problem [4, 5]. There are many research results obtained in recent years, but the recommendation courses are often based on the history of students' choice of courses and the big data analysis results of popular courses. Although the recommendation results are also practical, they lack the support for the whole learning process of learners and lack the recommendation in combination with the training scheme of the school.

Personalized recommendation technology has been widely used in various fields, such as news headlines, online videos, “we media” short video, and so on, which effectively solves the problem of information overload in various fields [6]. As the recommendation algorithm of knowledge graph as side information has been fully concerned and studied, it is possible for the courses recommendation algorithm based on knowledge graph to further improve the recommendation effect [7].

## 2. Overview of the Course Recommendation Algorithm

### 2.1. The Course Recommendation Algorithm

Course personalized recommendation technology is one of the research hotspots in the field of online education and big data education. Many research teams all over the world have put forward the concept of personalized online education and studied personalized course recommendation algorithms in order to reduce the dropout rate of online learning, stimulate and mobilize learners' enthusiasm for active learning, and give full play to the learning personality of different learners.

Bhaskaran and Santhi mined learners' browsing logs according to learners' behaviour and preferences and used the hybrid recommendation strategy proposed by ApriorAll algorithm to realize personalized recommendation for learners [8]. Based on the traditional data mining methods, Obeidat et al. [9] and others used collaborative filtering method and comparison of association rules to recommend courses for learners. The experimental results showed that grouping and clustering learners can significantly improve the recommendation effect. Huang et al. used reinforcement learning method and Markov decision process [10] to recommend exercises for learners. When recommending, they comprehensively considered the smoothness of exercise difficulty, review and preview, and the degree of learners' participation. Liu et al. proposed using the method based on neural network [11] to track learners' knowledge level, so as to provide learners with personalized learning path recommendation. The MCRs proposed by Zhang et al. are based on the distributed association rule mining algorithm [12], which makes the recommendation information transmitted more timely and improves the user's course retrieval efficiency.

Chen et al. carried out a knowledge recommendation algorithm based on learners' existing knowledge and learning materials [13], which modelled the recommendation process as a Markov decision problem. Aguilar et al. analysed the differences and similarities between the course selection system and the e-commerce platform, improved knowledge, and discovered association rules through biological heuristic algorithms, while most of the research in recent years were to explore the behaviour characteristics of learners to represent the characteristics of learners, so as to produce recommendation results [14].

Pang et al. extracted the behaviour characteristics of each learner and converted them into vectors of the same dimension, and dispersed them among similar users, so these learners would have more same courses [15]. They proposed multilayer bucket recommendation and MapReduce technology extension, so as to increase the satisfaction of the traditional collaborative recommendation algorithm.

To sum up, there have been a lot of works in learner personalized course recommendation recently years.

### 2.2. The Problems in the Traditional Course Recommendation Algorithm

At present, the existing research works mainly focused on using collaborative recommendation or data mining methods to improve the accuracy of course recommendation, but there are some problems in these methods, such as the cold start of recommendation algorithm, building a recommendation model without good performance, and carrying out an algorithm which cannot accurately recommend without initial data [16]. These problems are described as follows:

1. Accuracy of course recommendation: the recommendation algorithm using highly accurate rate can provide learners with more suitable courses and enhance their satisfaction. The accuracy of recommendation results is an important index of recommendation algorithm. If the recommendation result generated by a recommendation algorithm does not bring out good recommendation results, the recommendation results will be meaningless in some certain [17].
2. The problem of data sparsity: when using the traditional collaborative filtering method, the similarity calculation mainly depends on the learners' rating matrix of the course. In the actual process, the number of the course rating score is very small; in some cases, the scoring users account for only 2% of the total users. It makes the course scoring matrix so sparse, and it is difficult for courses and learners to find similar neighbors, resulting in the low quality of courses recommendation [18].
3. Cold start problem: now the course platform would constantly update the content under the needs of new learners and new courses, but there is no record of new learners or new course content before updating the course platform, resulting in the failure of the recommendation algorithm to make timely and effective recommendations [19].

## 3. Related Research

### 3.1. Recommendation Based on Collaborative Filtering

Collaborative filtering algorithm is widely used in recommendation field. Traditional recommendation algorithms ignore the characteristic data of users or items in advance and only rely on the historical behaviour data of users to build models and recommend items to users. In most collaborative filtering algorithms, nearest neighbor technology is used to calculate the distance between users with the help of historical preference information [20]. The algorithm uses the weighted score of the nearest neighbor of the target user to predict the target user's preference for specific courses, but it often faces problems such as sparse data and mining interpretation of recommendation results.

Collaborative filtering has been successfully applied in different researching fields [21]. The collaborative filtering recommendation system is based on the idea that similar users may have similar preferences or similar items may be preferred by similar users [22]. Collaborative filtering is generally divided into two categories:

1. User-based algorithm [23]: according to the users' behaviour record, it would find the user set which is similar in the interest and preference of the target user, then find the items liked by each user in this user set, filter out the items that the target user has already selected, and finally recommend the remaining items to the target user. In this method, the user similarity matrix calculated by the similarity formula is used to predict the target user's score on the items liked by similar users.
2. Item-based algorithm [24]: according to the users' behaviour records, it calculates the similarity between items and filters out the item set with high score among the item sets generated by the target user. Then, according to the item similarity matrix, the other items, most similar to each item in the collection, would be found out, sorting and filtering the items that the target user has selected at the same time, and then finally the rest items are recommended to the target user.

The collaborative filtering algorithm uses the weighted score of the nearest neighbor of the target user to predict the target user's preference for specific courses, but sometimes it would face the problems of sparse data and unexplained recommendation results.

### 3.2. Recommendation Method Based on Knowledge Graph

Google put forward the concept of knowledge graph, which uses the “entity-relation-entity” 3-tuple to describe the semantic relationship between different entities in the real world and forms a network knowledge structure through the relationship [25]. Representation learning aims to transform the objects into the low-dimensional space [26], and the aim of knowledge graph representation learning is to map the entities and relationships in the knowledge graph. The resulting vector can effectively represent the semantic relationship between entities and relationships [27].

Recently, the representation learning methods of deep learning have attracted extensive attention [28–34]. The common models of knowledge graph representation learning include distance model, energy model, matrix decomposition model, bilinear model, translation model, and so on [35]. The translation model represented by Ebisu and Ichise [36] has fewer parameters and is more popular in the research fields. For each 3-tuple *(h*, *r*, *t*), where *h* and *t* represent head entities and tail entities, respectively, *r* is the relationship between them, TransE represents *h*, *t*, and *r* as the embedded vectors *v*_*h*_, *v*_*t*_, and *v*_*r*_, respectively, and *v*_*r*_ is the translation from vectors *v*_*h*_ to *v*_*t*_, and the relationship among the three elements is expressed as follows:

(1) $$\begin{array}{c} v_{h}+v_{r}\approx v_{t}. \end{array}$$

In the TransE model, it aims to train vectors *v*_*h*_, *v*_*t*_, and *v*_*r*_ effectively to make formula (1) infinitely close to be equal. The smaller the error between them, the more likely there is a relationship *r* between the head and tail. Therefore, the loss function is shown as follows:

(2) $$\begin{array}{c} f\left(v_{h},v_{r},v_{t\;}\right)={\left\|v_{h}+v_{r}-v_{t\;}\right\|}_{2}^{2}, \end{array}$$

where ‖·‖_2_ is the 2-norm of the vector, that is, the Euclidean distance. The total cost function for all 3-tuple samples is expressed as follows:

(3) $$\begin{array}{c} L=\sum_{\left(h,r,t\right)\in S}\sum_{\left(h^{\prime },r,t^{\prime }\right)\in S^{\prime }}\max\left(0,f\left(v_{h},v_{r},v_{t\;}\right)-f\left(v_{h^{\prime }},v_{r},v_{t^{\prime }\;}\right)+\delta \right), \end{array}$$

where *S* is positive sample and is the set of all 3-tuple in the knowledge graph; *S′* is the negative samples of *S*, that is, each existing 3-tuple in *S* is randomly replaced with its head entity or tail entity to obtain a new 3-tuple, and the 3-tuple does not belong to *S*; then the set of such 3-tuples is called negative sample; and *δ* is the distance between *S* and *S'*. In order to minimize the cost function *L* during training process, it is necessary to make the loss function *f*(*v*_*h*_, *v*_*r*_, *v*_*t* _) of positive samples tend to *0* and the loss function *f*(*v*_*h*′_, *v*_*r*_, *v*_*t*′ _) of negative samples tend to infinity.

## 4. Recommendation Algorithm Fusing with Knowledge Graph and Collaborative Filtering Technology

### 4.1. The Algorithm Model

**Figure 1:**
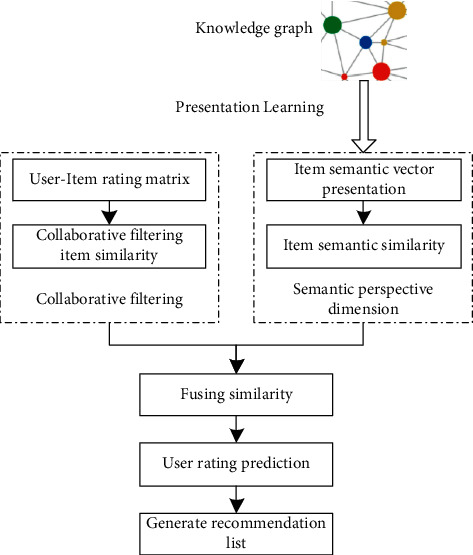
FKGCF algorithm model.

At present, collaborative filtering recommendation algorithms usually use users' historical evaluation data without considering the semantic relationship between recommendation items. To solve this problem, based on previous studies, a courses resource recommendation algorithm fusing knowledge graph and collaborative filtering technology (FKGCF) was proposed. This algorithm directly integrates the semantic similarity of the recommended object into the similarity calculation of the collaborative filtering recommendation algorithm and makes up for the deficiency that this algorithm ignores the connotation characteristics of the item itself from the semantic perspective. The algorithm model is shown in Figure 1.

### 4.2. Composition of Course Knowledge Graph

The interaction information between learners and courses in the existing data sets is sparse or even missing, which will lead to the cold start problem. This problem can be alleviated by introducing other information, namely, side information, into the recommendation algorithm. Side information introduced in common recommendations such as movies and commodities includes social networks, user/item attributes, multimedia, and contexts [4]. The side information used in this paper is the knowledge graph, which is represented by 3-tuple (*C*_1_, *R, C*_2_). The knowledge graph of the course is *G*:

(4) $$\begin{array}{c} G=\left\{\left(c_{1},\;r,\;c_{2}\right)|c_{1},\;c_{2}\in C,\;\;r\in R\right\}, \end{array}$$

where *c*_1_,  *c*_2_ ∈ *C*, *C* is the set of all the courses, and *r* ∈ *R*; there are 5 relations in *R*: same.instructor, same.subject, hour.low, hour.mid, and hour.high. When a learner chooses some certain courses, these courses can be connected to other events in the knowledge graph, connected to many other nonitem entities, and then connected to other course entities from these nonitem entities. For example, if there are computer courses in the selected courses, it can be related to other computer courses belonging to the same subject. Then, a 3-tuple knowledge graph is built.

In the traditional collaborative filtering algorithm, the connection is established by other learners' historical learning records and other interactive processes. The essence of the knowledge graph constructed by the FKGCF algorithm is to establish a connection between the courses which learners have interacted and the courses which they have not interacted. These connections are not obtained from the interaction history of other learners, but through non-item entities. The method of constructing knowledge graph provides additional information sources connected between courses and a more accurate calculation method of item similarity in the algorithm, so as to improve the accuracy of recommended courses [37].

### 4.3. The Semantic Similarity

The entities and relationships in the knowledge graph are embedded into a d-Dimensional semantic space by using the TransE algorithm, and the item semantic vector is expressed as follows:

(5) $$\begin{array}{c} I_{i}={\left(E_{1i},E_{2i},\ldots ,E_{\text{di}}\right)}^{T}, \end{array}$$

where *I*_*i*_ presents the semantic vector of item *I* and *E*_ki_ is the value of the *k*th dimension semantic vector, where 1 ≪ *k* ≪ *d*. The TranseE algorithm trains the loss function based on Euclidean distance. The similarity of item semantics is also measured by this distance, and the calculation equation is as follows:

(6) $$\begin{array}{c} d\left(I_{i},I_{j}\right)=\sqrt{\sum_{k=1}^{d}{\left(E_{\text{ki}}-E_{\text{kj}}\right)}^{2}}. \end{array}$$

In order to unify the value of distance within the range of (0, 1), the following calculation method is carried out:

(7) $$\begin{array}{c} {\text{sim}}_{\text{sem}}\left(i,j\right)=\frac{1}{1+d\left(I_{i},I_{j}\right)}. \end{array}$$

The greater the value of sim_sem_(*i*, *j*) is, the more similar the semantics of items *i* and *j* is.

In the knowledge graph, these pieces of feature information form a “entity-relationship-entity” 3-tuple. The more similar the semantic information is, the closer the course vector is, and the more similar the users' love for it. Using these rich semantic data, the cold start problem of recommendation system is solved to a certain extent.

### 4.4. Fusing Similarity

After the item semantic similarity sim_sem_ was obtained by knowledge graph representation algorithm, the similarity in the collaborative filtering recommendation algorithm was calculated, as shown in

(8) $$\begin{array}{c} {\text{sim}}_{\text{CF}}\left(i,j\right)=\cos\left(S_{i},S_{j}\right)=\frac{S_{i}\cdot S_{j}}{\left\|S_{i}\right\|\cdot \left\|S_{j}\right\|}=\frac{\sum_{k=1}^{m}r_{\text{ki}}\cdot r_{\text{kj}}}{\sqrt{\sum_{k=1}^{m}r_{\text{ki}}^{2}}\cdot \sqrt{\sum_{k=1}^{m}r_{\text{kj}}^{2}}}, \end{array}$$

where sim_CF_(*i*, *j*) is the cosine similarity of collaborative filtering of items *i* and *j*, and *S*_*i*_ and *S*_*j*_ are the user's scoring vectors for items *i* and *j*, respectively.

Next, we fuse two similarities, sim_CF_(*i*, *j*) and sim_sem_(*i*, *j*), as shown in

(9) $$\begin{array}{c} \text{sim}\left(i,j\right)=\alpha \cdot {\text{sim}}_{\text{sem}}\left(i,j\right)+\left(1-\alpha \right)\cdot {\text{sim}}_{\text{CF}}\left(i,j\right), \end{array}$$

where sim(*i*, *j*) represents fusing similarity of item *i* and *j*, sim_sem_(*i*, *j*) is the items semantic similarity, sim_CF_(*i*, *j*) is the collaborative filtering item similarity, and *α* is the weighted factor, whose value range is [0, 1]. When *α* is 0, the algorithm is based on items. When *α* is 1, the algorithm is based on semantic. When *α* ∈ (0,1), it is the fusing recommendation algorithm. The collaborative filtering recommendation algorithm is to calculate the similarity of items indirectly from the perspective of users' rating of items.

### 4.5. Rating Prediction and Generation of Recommendation List

According to the fusing similarity, the user's score on an item that has not been evaluated before is predicted. The calculation equation of prediction score is as follows:

(10) $$\begin{array}{c} p_{\text{ui}}=\frac{\sum_{j\in \left(N\left(u\right)\cap S\left(i,k\right)\right)}\left(\text{sim}\left(i,j\right)*r_{\text{uj}}\right)}{\sum_{j\in \left(N\left(u\right)\cap S\left(i,k\right)\right)}\text{sim}\left(i,j\right)}, \end{array}$$

where *p*_ui_ represents the prediction score that user *u* gives to item *i*. *N* (*u*) refers to all item sets evaluated by user *u*, *S* (*i*, *k*) refers to *k* adjacent items with the greatest similarity to item *i*, *N* (*u*)∩*S* (*i*, *k*) refers to the intersection of two item sets, and the intersection result is the reference for prediction score; sim (*i*, *j*) is the fusing similarity of items *i* and *j*; r_uj_ is the user *u*'s rating of item *j.*

The higher the prediction score is, the more interested the user is. The prediction score of all items is calculated by using equation (10). And then they are sorted according to the scoring results, giving priority to the top-*N* items recommended to the user.

## 5. Experimental Process

### 5.1. Experimental Environment and Data Set Processing

The experimental data comes from XuetangX. 1000 courses and 10000 members are collected for experiments. The precision, recall, and F1 value are used as evaluation indexes to measure the performance of each algorithm. The precision refers to the probability that the user is interested in the recommended course list, and recall refers to the probability that the course that the user is interested in appears in the recommendation list. F1 value is the harmonic average of precision and recall [38]. The calculating methods are shown as follows:

(11) $$\begin{array}{c} \text{Precision}=\frac{\text{TP}}{\text{TP}+\text{FP}},\\ \text{Recall}=\frac{\text{TP}}{\text{TP}+\text{FN}},\\ F1=\frac{2\times \text{Precision}\times \text{Recall}}{\text{Precision}+\text{Recall}}. \end{array}$$

In order to measure the performance of the algorithm more accurately, this paper uses *k*-cross verification and the *k* value is 5; that is, the experimental data are randomly grouped in 5 parts, one of which is selected as the testing set and the other 4 as the training set. A total of 5 tests are carried out, and the average result of 5 tests is used as the final evaluation of this algorithm.

### 5.2. Experimental Process and Result Analysis

#### 5.2.1. Experiment of Different Embedding Dimensions

**Figure 2:**
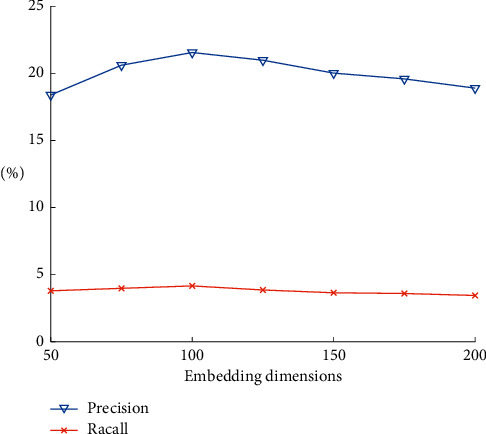
Results' comparison under different embedding dimensions.

When knowledge graph representation learning is carried on, different embedding dimensions will have different results in the experiment. Seven dimensions, 50, 75, 100, 125, 150, 175, and 200, are selected for the experimental comparison. The similarity fusing weight factor *α* is set to 0.6, and the maximum number of neighbors *k* of similarity is set to 30. The final experimental comparison results are shown in Figure 2.

From Figure 2, it can be found that there is no significant difference among the seven different embedding dimensions. But the precision and recall value are better when the dimension is 100.

#### 5.2.2. Experiment of Different Fusing Weight Factor Ratio

**Figure 3:**
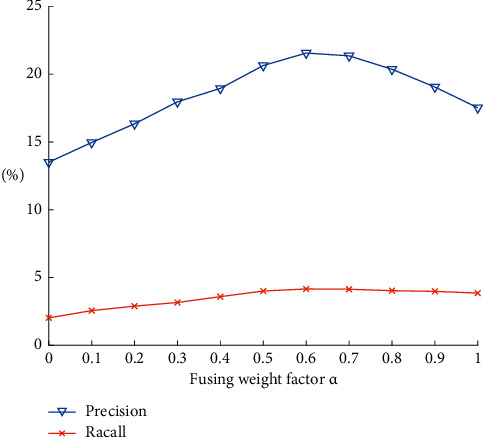
Results' comparison under different *α* values.

Fusing weight factor *α* controls the proportion of semantic similarity and collaborative filtering similarity in the final fusing similarity, and it is a key factor of this algorithm. The value range of *α* is [0, 1], and in the experiment *α* is assigned from 0 to 1 with a step of 0.1, so there are 11 steps altogether. The embedding dimension of knowledge graph presentation learning is set to 100, and the maximum number of neighbors *k* of similarity is set to 30. The results can be seen in Figure 3.

Experiments show that when the fusion reaches a certain proportion, the precision and recall of the proposed algorithm are higher than the single semantic content-based recommendation algorithm (*α* = 1) and the item-based algorithm (*α* = 0). When the fusion weight *α* is 0.6, the precision and recall reach the best value.

#### 5.2.3. Comparison of Experimental Results

**Figure 4:**
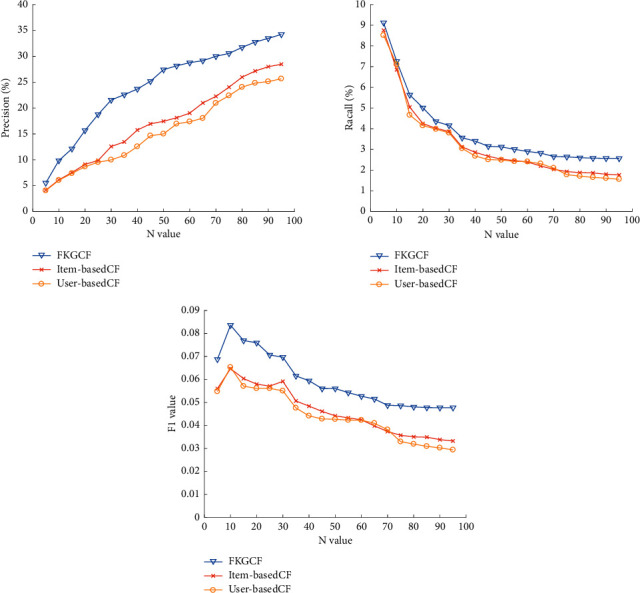
Experimental results (precision, recall, and F1 value).

To verify the performance of the modified collaborative filtering recommendation algorithm proposed in this paper, experimental comparisons are made with traditional algorithms, user-basedCF and item-basedCF. The embedding dimension of knowledge graph representation learning in this algorithm is set to 100. The fusing weight *α* is set to 0.6 and the recommended number top-*N* is set to 10. The experimental results are shown in Figure 4. The *N* value refers to the number of neighbors in the nearest neighbor algorithm KNN. The figure illustrates that the precision of all comparative experiments increases with the increase of *n* value while the recall decreases, and the F1 value increases first and then decreases.

Through experimental comparison, it is obvious that the proposed FKGCF algorithm has higher precision, recall, and F1 value than the traditional algorithms.

## 6. Conclusions

Aiming at the problem that the collaborative filtering recommendation algorithm only uses the users' historical evaluation data without considering the semantic relationship between the recommended objects, a modified FKGCF algorithm was proposed. The algorithm not only uses the users' evaluation information of the course, but also uses the internal semantic information of the item itself. The results show that this algorithm improves the precision and recall and can realize the courses recommendation more accurately and efficiently. However, the algorithm in this paper also has some limitations, which does not consider the problem of user interest drift, because the users' interest is likely to change with the development of time, and the previous historical data is time-effective. Therefore, according to the time dynamics of historical data, establishing relevant time series recommendation models is a direction worthy of further research.

## Data Availability

The data used to support the findings of this study are available from the corresponding author upon request.

## References

[1] China Internet Network Information Center (CNNIC) (2020). *The 46th Statistical Report on the Current Situation of Internet Development in China*.

[2] Xiao J., Wang M., Jiang B., Li J. (2018). A personalized recommendation system with combinational algorithm for online learning. *Journal of Ambient Intelligence and Humanized Computing*.

[3] Zhang H., Yang H., Huang T., Zhan G. DBNCF: personalized courses recommendation system based on DBN in MOOC environment.

[4] Yang C., Bai L., Zhang C., Yuan Q., Han J. Bridging collaborative filtering and semi-supervised learning: a neural approach for poi recommendation.

[5] Wen J., Wei X., He T., Zhang S. (2020). Regression analysis on the influencing factors of the acceptance of online education platform among college students. *Ingénierie des Systèmes d’Information*.

[6] Zhang Q., Liu Y., Liu L., Lu S., Feng Y., Yu X. (2021). Location identification and personalized recommendation of tourist attractions based on image processing. *Traitement du Signal*.

[7] Xian Y., Fu Z., Muthukrishnan S., De Melo G., Zhang Y. Reinforcement knowledge graph reasoning for explainable recommendation.

[8] Bhaskaran S., Santhi B. (2019). An efficient personalized trust based hybrid recommendation (tbhr) strategy for e-learning system in cloud computing. *Cluster Computing*.

[9] Obeidat R., Duwairi R., Al-Aiad A. A collaborative recommendation system for online courses recommendations.

[10] Huang Z., Liu Q., Zhai C. Exploring multi-objective exercise recommendations in online education systems.

[11] Liu Q., Tong S., Liu C. Exploiting cognitive structure for adaptive learning.

[12] Zhang H., Huang T., Lv Z., Liu S., Zhou Z. (2018). MCRS: a course recommendation system for MOOCs. *Multimedia Tools and Applications*.

[13] Chen Y., Li X., Liu J., Ying Z. (2018). Recommendation system for adaptive learning. *Applied Psychological Measurement*.

[14] Aguilar J., Valdiviezo-Díaz P., Riofrio G. (2017). A general framework for intelligent recommender systems. *Applied Computing and Informatics*.

[15] Pang Y., Jin Y., Zhang Y., Zhu T. (2017). Collaborative filtering recommendation for MOOC application. *Computer Applications in Engineering Education*.

[16] Liu Y., Yang H., Sun G., Bin S. (2020). Collaborative filtering recommendation algorithm based on multi-relationship social network. *Ingénierie des Systèmes d’Information*.

[17] Lin J., Pu H., Li Y., Lian J. (2018). Intelligent recommendation system for course selection in smart education. *Procedia Computer Science*.

[18] Tian Y., Zheng B., Wang Y., Zhang Y., Wu Q. (2019). College library personalized recommendation system based on hybrid recommendation algorithm. *Procedia CIRP*.

[19] Gope J., Jain S. A survey on solving cold start problem in recommender systems.

[20] Shen J., Zhou T., Chen L. (2020). Collaborative filtering-based recommendation system for big data. *International Journal of Computational Science and Engineering*.

[21] Subramaniyaswamy V., Logesh R., Chandrashekhar M., Challa A., Vijayakumar V. (2017). A personalised movie recommendation system based on collaborative filtering. *International Journal of High Performance Computing and Networking*.

[22] Sattar A., Ghazanfar M., Iqbal M. (2017). Building accurate and practical recommender system algorithms using machine learning classifier and collaborative filtering. *Arabian Journal for Science & Engineering*.

[23] Tan Z., He L. (2017). An efficient similarity measure for user-based collaborative filtering recommender systems inspired by the physical resonance principle. *IEEE Access*.

[24] Thakkar P., Varma K., Ukani V., Mankad S., Tanwar S. (2019). Combining user-based and item-based collaborative filtering using machine learning. *Information and Communication Technology for Intelligent Systems*.

[25] Jia B., Huang X., Jiao S. (2018). Application of semantic similarity calculation based on knowledge graph for personalized study recommendation service. *Educational Sciences: Theory & Practice*.

[26] Ebisu T., Ichise R. Toruse: knowledge graph embedding on a lie group.

[27] Fan M., Zhou Q., Zheng T. F., Grishman R. (2017). Distributed representation learning for knowledge graphs with entity descriptions. *Pattern Recognition Letters*.

[28] Xu K., Li C., Tian Y., Sonobe T., Kawarabayashi K. I., Jegelka S. Representation learning on graphs with jumping knowledge networks.

[29] Xu G., Li X., Zhang Z. (2020). Semantic consistency cross-modal retrieval with semi-supervised graph regularization. *IEEE Access*.

[30] Meng W. L., Mao C. Z., Zhang J., Wen J., Wu D. H. (2020). A fast recognition algorithm of online social network images based on deep learning. *Traitement du Signal*.

[31] Çinar A., Yildirim M. (2020). Classification of malaria cell images with deep learning architectures. *Ingénierie des Systèmes d’Information*.

[32] Doppala B. P., Bhattacharyya D., Chakkravarthy M. (2020). Stratification of cardiovascular diseases using deep learning. *Revue d’Intelligence Artificielle*.

[33] Xu G., Li X., Shi L., Zhang Z., Zhai A. (2020). Combination subspace graph learning for cross-modal retrieval. *Alexandria Engineering Journal*.

[34] Demircan S., Örnek H. K. (2020). Comparison of the effects of mel coefficients and spectrogram images via deep learning in emotion classification. *Traitement du Signal*.

[35] Lin Y., Han X., Xie R., Liu Z., Sun M. (2018). Knowledge representation learning: a quantitative review. https://arxiv.org/abs/1812.10901.

[36] Ebisu T., Ichise R. (2019). Generalized translation-based embedding of knowledge graph. *IEEE Transactions on Knowledge and Data Engineering*.

[37] Zarzour H., Al-Sharif Z., Al-Ayyoub M., Jararweh Y. A new collaborative filtering recommendation algorithm based on dimensionality reduction and clustering techniques.

[38] Xu G., Zhai A., Wang J., Zhang Z., Li X. (2019). Cross-media semantic matching based on sparse representation. *Technical Gazette*.

